# A Eugenic View of the Emancipation of Woman

**Published:** 1912-10

**Authors:** 


### A Eugenic View of the Emancipation of Woman.

BY A WIFE AND MOTHER.

Those of us who had the privilege of knowing our grandparents well remember the prophecy of the old people that, "in the latter days there shall be wars and rumors of wars, pestilence, plagues," etc., trying to point out that at that period the millennium was approaching.

According to the "signs of the times," we *are,* and have been, in the "latter days," for we are also told that "a thousands years are as a day" in the sight of the Infinite. And we have had wars, and rumors of wars, and pestilence, and scourges, and not the least of these is the unsettled condition of our social system today.

Anything which undermines our corner-stone (the home) may surely regarded as a "plague" or "scourge."

Woman was made for *man,* not man for *woman!* Yet, to note existing conditions, one would think that nature was revolting and trying to reorganize herself.

Man was made *first,* and in the Divine image, and pronounced "good," a being imbued with qualities of mind and body, with desires and appetites, to be satisfied, yet incomplete, with all; for *within him* was the lack of ability to satisfy these same cravings or desires; so he must have a mate, a counterpast, a consort, if you please; in other words, he must have an outlet for his passions, his cravings, his desires, and, as in the animal world, the mate, or female, was made to meet the requirements of the brute creation and to fulfill the law of nature (procreation or reproduction of the species); so man must have his mate, his affinity.

Thus *woman* came to be!

Man has two appetites: the animal or physical and the mental or spiritual.

Both demand gratification, for both are insistent of relief. If the answer of these may be found in *one* woman, and that one be the *wife,* well and good. But, alas! too many women feed only one side of a husband's nature, starving the other the while, which accounts for polygamy in primeval man and which is still extant in some parts of the world today.

Thus you can see that "a man may love two women at the same time."

If wives could realize this, would they not try to be the "all-round woman," as some one has expressed it? I think so.

Too many wives are "one-sided," by allowing "children," "domestic duties," or "social" or "club" life to bias their natures, thus losing that hold upon their husbands which the "all-round" wife or woman will never do.

Our social system has imposed monogamy on man, and yet we wives make it just as hard as we can for the poor fellows, instead of being *true helpmates.*

When a man marries, he wants a companion, not a housekeeper, nor a slave, nor a nurse girl; but a wife, a comrade, a chum!

Women, you who are now feeling lonely, and neglected, and clamoring for your "rights," remember that when "he" courted you he thought he saw in you his ideal, and so he selected you, out of hundreds, and by your own attitude today you are proclaiming to the world that *he* was mistaken, that you were not what you seemed; that the little artifices and coquettish dependencies you assumed, to capture him, were "only acting."

You forget that man lives in the practical, material side of life, while we women live a little too much in the psychical; in many instances, entirely so.

While nature gave to woman a finer temperament, more psychical and sensitive and impractical in a way, yet it was intended so that the union of the sexes would thus make a complete whole. In other words, that part of nature which was lacking in *man* was given to *woman,* that she might be his counterpart or complement. Otherwise, men would prefer the society of their own sex, finding it all sufficient.

Thus it transpires that it is woman's *dependence,* her weakness, her finer nature, which makes her attractive to man, developing the best in him, causing him to assume his natural attitude toward her sex, viz.: one of defender or protector..

As a girl, I remember hearing several men referred to as "Miss Nancy's." Why? Because they had invaded woman's sphere, and, if not actually assuming the apron (her badge of office), had virtually decided to run the house, doing even the housework. In the proportion of their assumption of her sphere, they were regarded with contempt, and their wives were pitied, for, in the ratio of their descent from their proper sphere—that of *wage-earner* and defender and protector—they were supposed to lose much of their noble, defensive qualities, and so were objects of derision and contempt. For man is ever attractive to woman, as he appeals to her nature, as something stronger, larger and more noble than herself; someone to whom she may look up for care, protection, and advice.

On the other hand, with a man, it is the sweetheart to whom he applies the term "dear little girl," that he asks to be his wife, for it is she who calls out of him the *parental,* or best side of his nature.

The cry we hear on every hand is "more power," "larger opportunities" and "equal rights."

Was it not woman's subtle power that had John the Baptist beheaded? Whose intuition was it which devised a way to take a Samson's strength? So much for the power of the *evil* woman.

On the other hand. was it the rod and scepter, or the subtle influence of woman, which made Queen Esther the redeemer of her people.

And so it has ever been. Woman's power has lain in her intuitive knowledge and subtle way of "knowing how" to attain the accomplishment of her desires, and "not by might, but by right," was her victory gained or the battle won—*the right of "knowing how."* Women are born diplomatists, and Webster says, "diplomacy is dexterity or skill in securing advantages," or the "science or art of conducting negotiations between nations, particularly in securing treaties," etc.

Now, in the light of the twentieth century, when war or warfare is considered a relic of barbarism, and difficulties between nations are decided in a court of arbitration, how can enlightened women of our time, who are in possession of their faculties and mistresses of a science as powerful as diplomacy, step *down* from their throne of dignified wifehood and glorious motherhood and resort to lower or physical means of accomplishing any purpose? Why it is virtually acknowledging to the world their own lack of power. Woman, being a finer organism than man and living more in the psychical sphere than he, has fallen heir to this art, and should cherish it as a divine gift, and use it for man's assistance and advancement and not for his degradation or abasement. As man is aggressive by nature, so is woman submissive. If we had not been born so, the human race would have become extinct eons ago. For not only would man have continued to draw his bow against his brother, but woman would have joined in the melée, and extinction of the race would have been the result.

This trait of "submission" being inborn, or inbued in woman, has been handed down to our day, and is still the key to race preservation. It is one of the unwritten laws of society for opposites to marry. Why? Not only for physical resultant reasons to progeny, but also for psychical reasons; for the maintenance of peace in the home, as typified in the old nursery rhyme we all know of Jack Sprat and his wife, exemplifying the true relation of husband and wife. There can be but *one* head of the household, the other must be a *subhead*, or else there will be friction. It is so in the business world, and society and club life; as soon as *two* try to run anything, then there is conflict or rupture. How necessary is it to recognize this, in the home sphere, when so much is at stake. The destiny of a nation may even be involved, for *our children* are the *men of tomorrow*.

Had you ever thought of that? and yet some of you cry for "larger opportunities."

It is indeed fortunate that nature has made the decision for us (that man should be the legal head of the family and "defender of the faithful"), for woman, by her finer organism, is not qualified to battle with the world and its evils.

Then, her natural sphere is so large that she has no time to engage in man's work, for she is doing something greater than attending to the petty vexations of an everyday business life, which resolves itself simply into a scramble for the Almighty dollar, with which to feed, house and clothe us *today*, while *she* has the privilege of building minds and forming character which shall build a nation or level an empire of *tomorrow*.

Isn't that worth something? Could any opportunity be larger or grander or more noble?

Then, if she is so sheltered and blessed with health and strength as to live to see the fruition of her labor, then when she comes to life's swift closing gate may she not compare her opportunities and results with her husband, and say, with Simeon of old, "Lord, now let Thy servant depart in peace."

The last and loudest of the protests or cry of the hour is "equal rights" or the "emancipation of woman."

Now, here again, women are proclaiming to the world their own lack of power, their lack of ability to "make good." There are physical reasons involved here.

Let me ask you, is woman's temperament or organism or are bodily functions any different, or do they work on any more advanced lines today, than they did for Eve? I think not.

Well, the world through the law, it being "man-made," recognizes these things, and with all due regard to woman's physical limitations has arranged the laws accordingly.

Of course, we all know the physical reason which makes hardships or undue exposure during an entire thirty days undesirable for woman, and any physician can explain the physical necessity for rest, for lack of *mental* strain at such a time, and the resultant effects of "nervousness" attendant upon infraction of these natural laws. And there is another reason. This desire for a "larger sphere," or more "rights," has its origin in the sex zone. "Horrid!" you say. Well, listen!

Woman has two natures, as has man, but her appetites do not mature as do his. *He* is animal first and then psychic; she is psychic and then animal. In other words, by training and environment, her animal or physical nature is kept down or under control, until after marriages, when it must then be aroused, even educated.

This process sometimes takes years, so that a woman may even arrive at middle life before she recognizes it, or the value of it to her nature.

Man arrives at the appreciation or value of his sexual nature much earlier than woman, and it might be said to never know diminution, unless attacked by disease or extreme old age.

The average wife, after fifteen years or more of married life, especially if there have been children, has probably lost that stronghold on her husband's affections which was hers by right, and sexually they have drifted apart.

Here is the "soft brick" in the superstructure of marriage which is the keystone to happiness.

For, with her arrival at this health zone, which is between thirty-five and fifty (and with care and health this may safely be extended ten years), she is confronted with the deplorable situation just depicted, and is now with the full consciousness of her powers upon her, and is thrown upon her own resources to meet the situation.

Being a virtuous woman, this demand of her nature is now filled or supplied *mentally* by her psychical force. With this power she now diverts the hitherto dormant forces of her nature to "mothering the world" or "harboring humanity," which finds expression in the pursuit of the *arts, literature,* club, or social life.

For now it is that all the faculties are kindled, her finer sensibilities quickened, and her perceptions are keener. In other words, she comes into this new zone of her being with all the fullness of her nature, and it is at this time that she may make the best wife, the most adorable mother, the most brilliant writer, the great society leader, or the efficient club woman.

For the woman of thirty-five to fifty has come into her own, as her early training was to suppress all sex radiations, and her early married life and child-bearing period probably stultified it, but now she is a "new woman" indeed; being fully matured, she understands life better, and understanding life means the fullest realization of love, and the fullest realization of love means the keenest appreciation of man.

She has been many years being educated up to this point, and, during all that period, someone has stood between the pitiless world and hunger and despair, and patiently toiled for her welfare. Now, in the light of her full knowledge, and the joy of her being and in the interest of "equal rights," should she not reward him for his deprivation and patience? Does she not *owe* him something? If she believes so, then, indeed, will she be an

"all-round woman," for she will be *emancipated through her interpretation of the thought* that *Life* is *Love*, and *Love* is *Life* and *Light* and *Joy!*

